# {2-(4-Hy­droxy­phen­yl)-2-[(3-meth­oxy-2-oxidobenzyl­idene)amino-κ^2^ *O* ^2^,*N*]propanoato-κ*O*}(1,10-phenanthroline-κ^2^ *N*,*N*′)copper(II) dihydrate

**DOI:** 10.1107/S1600536811009627

**Published:** 2011-03-19

**Authors:** Xuewei Pu, Lianzhi Li, Jianfang Dong, Buqin Jing

**Affiliations:** a School of Chemistry and Chemical Engineering, Liaocheng University, Shandong 252059, People’s Republic of China; b Department of Material Science, Shandong Polytechnic Technician College, Shandong 252027, People’s Republic of China

## Abstract

In the title complex, [Cu(C_17_H_15_NO_5_)(C_12_H_8_N_2_)]·2H_2_O, the central Cu^II^ ion is five-coordinate, bound to one N atom and two O atoms from the Schiff base ligand and by two N atoms from a 1,10-phenanthroline ligand in a distorted square-pyramidal configuration. In the crystal, inter­molecular O—H⋯O and C—H⋯O hydrogen bonds form a two-dimensional network parallel to (001).

## Related literature

For background to Schiff bases and the applications of Schiff base–copper complexes, see: Chohan *et al.* (1998 ▶); Nath *et al.* (2001 ▶); Raso *et al.* (1999 ▶); Yamada (1966 ▶). For the structure of a similar complex with a five-coordinate Cu^II^ ion, see: Qiu *et al.* (2008 ▶).
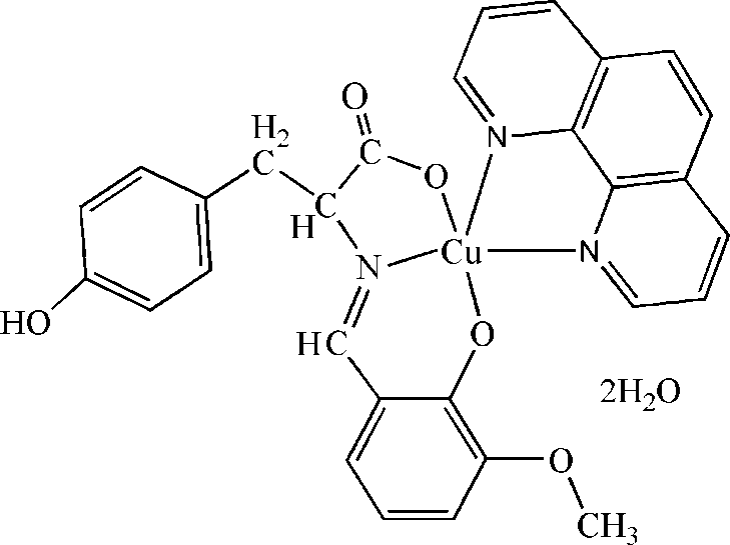


## Experimental

### 

#### Crystal data

[Cu(C_17_H_15_NO_5_)(C_12_H_8_N_2_)]·2H_2_O
*M* *_r_* = 593.08
Monoclinic, 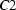

*a* = 11.755 (3) Å
*b* = 20.653 (5) Å
*c* = 13.202 (3) Å
β = 96.935 (4)°
*V* = 3181.6 (14) Å^3^
*Z* = 4
Mo *K*α radiation
μ = 0.73 mm^−1^
*T* = 298 K
0.48 × 0.42 × 0.38 mm

#### Data collection

Bruker SMART 1000 CCD area-detector diffractometer
Absorption correction: multi-scan (*SADABS*; Sheldrick, 1996 ▶) *T*_min_ = 0.720, *T*_max_ = 0.769
8117 measured reflections
3778 independent reflections
2811 reflections with *I* > 2σ(*I*)
*R*_int_ = 0.040

#### Refinement

*R*[*F* ^2^ > 2σ(*F* ^2^)] = 0.071
*wR*(*F* ^2^) = 0.202
*S* = 1.04
3778 reflections
363 parameters
15 restraints
H-atom parameters constrained
Δρ_max_ = 1.19 e Å^−3^
Δρ_min_ = −0.32 e Å^−3^
Absolute structure: Flack (1983 ▶), 956 Friedel pairs
Flack parameter: −0.02 (3)

### 

Data collection: *SMART* (Bruker, 2007 ▶); cell refinement: *SAINT* (Bruker, 2007 ▶); data reduction: *SAINT*; program(s) used to solve structure: *SHELXS97* (Sheldrick, 2008 ▶); program(s) used to refine structure: *SHELXL97* (Sheldrick, 2008 ▶); molecular graphics: *SHELXTL* (Sheldrick, 2008 ▶) and *DIAMOND* (Brandenburg, 1999 ▶); software used to prepare material for publication: *SHELXTL*.

## Supplementary Material

Crystal structure: contains datablocks global, I. DOI: 10.1107/S1600536811009627/rn2083sup1.cif

Structure factors: contains datablocks I. DOI: 10.1107/S1600536811009627/rn2083Isup2.hkl

Additional supplementary materials:  crystallographic information; 3D view; checkCIF report

## Figures and Tables

**Table 1:** Selected bond lengths (Å)

Cu1—N1	1.926 (10)
Cu1—O4	1.935 (5)
Cu1—O1	1.989 (5)
Cu1—N3	2.029 (9)
Cu1—N2	2.325 (8)

**Table 2:** Hydrogen-bond geometry (Å, °)

*D*—H⋯*A*	*D*—H	H⋯*A*	*D*⋯*A*	*D*—H⋯*A*
O6—H31⋯O2	0.85	1.93	2.684 (9)	148
O3—H3⋯O6^i^	0.82	2.53	2.843 (11)	104
O6—H30⋯O6^ii^	0.85	2.44	2.888 (11)	114
O7—H33⋯O2^iii^	0.85	2.15	2.721 (13)	125
C18—H18⋯O5^iv^	0.93	2.46	3.268 (13)	145

Symmetry codes: (i) 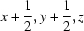; (ii) 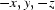; (iii) 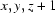; (iv) 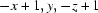.

**Fig. 1.**
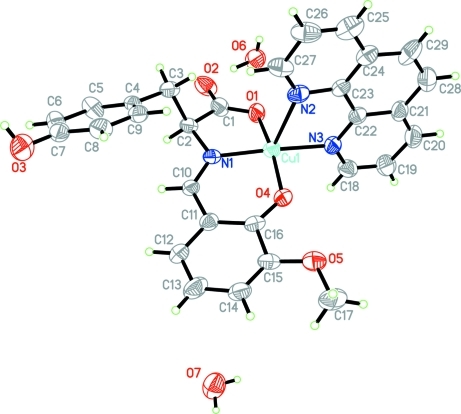
The structure of the title compound, drawn with 30% probability displacement ellipsoids.

**Fig. 2.**
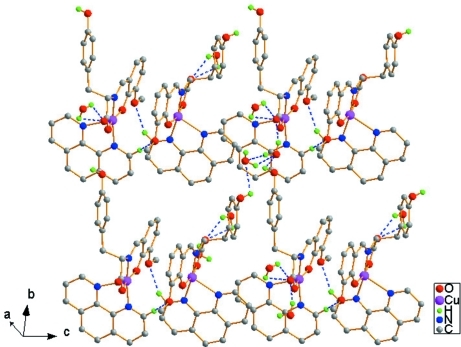
The two-dimensional network of the title complex linked by hydrogen bonds.

## supplementary crystallographic information

### Comment

Schiff bases still play an important role as ligands in metal coordination chemistry even after almost a century since their discovery (Yamada, 1966). It has been reported that amino acid Schiff bases and their first row transition metal complexes exhibit fungicidal, bactericidal, antiviral, and antitubercular activity (Chohan *et al.*, 1998; Nath *et al.*, 2001). Considerable efforts have been devoted to copper(II) complexes of tridentate *N*-alkylidene or *N*-arylidene alkanato Schiff base ligands, due to their structural diversity, electrochemical properties as well as a potential model for a number of important biological systems (Raso *et al.*, 1999). Herein, we report the synthesis and crystal structure of a new copper(II) complex with a tridentate Schiff base ligand derived from the condensation of *L*-tyrosine and *o*-vanillin, with a 1,10-phenanthroline coligand.

As shown in Fig 1, the central Cu^II^ ion is five coordinate, bound to two O atoms and one N atom of the Schiff base ligand and two N atoms of the 1,10-phenanthroline ligand, forming a distorted square-pyramidal geometry. The O1, O4, N1, and N2 atoms are in the equatorial plane, and N3 is in the axial position. The Cu^II^ ion lies 0.5068 (39)Å above the equatorial plane towards N3. The Cu1—N3 bond is significantly longer [2.325 (8) Å] (Table 1) as seen previously [2.231 (3) Å] (Qiu *et al.*, 2008).

In the crystal, the combination of intramolecular and intermolecular hydrogen bonds (Table 2) leads to a two-dimensional network (Fig. 2).

### Experimental

*L*-Tyrosine(1 mmol, 146.2 mg) and potassium hydroxide (1 mmol, 56.1 mg) were dissolved in hot methanol (10 ml) and added successively to a methanol solution of *o*-vanillin (1 mmol, 152.2 mg). The mixture was then stirred at 323 K for 2 h. Subsequently, an aqueous solution(2 ml) of cupric acetate monohydrate (1 mmol, 199.7 mg) was added dropwise and stirred for 2 h. A methanol solution (5 ml) of 1,10-phenanthroline (1 mmol, 198.2 mg) was added dropwise and stirred for 4 h. The solution was held at room temperature for ten days, whereupon green block-shaped crystals suitable for X-ray diffraction were obtained.

### Refinement

The maximum residual density peak in the final difference Fourier map is 1.189 e Å^-3^, it is 2.67Å and 4.018Å from H19 and Cu, respectively.

H atoms of the water molecules were found in difference Fourier maps and refined isotropically, with the O—H distances restrained to 0.85 (2)Å and with *U*_iso_(H) = 1.2*U*_eq_(O). All other H atoms were placed in geometrically calculated positions (C—H = 0.93 - 0.98 Å)and allowed to ride on their respective parent atoms, with *U*_iso_(H) = 1.2*U*_eq_(C_phenyl_) or 1.5*U*_eq_(C_methyl_) and O_hydroxyl_).

### Crystal data

**Table:**

[Cu(C_17_H_15_NO_5_)(C_12_H_8_N_2_)]·2H_2_O	*F*(000) = 1228
*M**_r_* = 593.08	*D*_x_ = 1.238 Mg m^−^^3^
Monoclinic, *C*2	Mo *K*α radiation, λ = 0.71073 Å
Hall symbol: C 2y	Cell parameters from 2405 reflections
*a* = 11.755 (3) Å	θ = 2.4–22.2°
*b* = 20.653 (5) Å	µ = 0.73 mm^−^^1^
*c* = 13.202 (3) Å	*T* = 298 K
β = 96.935 (4)°	Block, blue
*V* = 3181.6 (14) Å^3^	0.48 × 0.42 × 0.38 mm
*Z* = 4	

### Data collection

**Table:**

Bruker SMART 1000 CCD area-detector diffractometer	3778 independent reflections
Radiation source: fine-focus sealed tube	2811 reflections with *I* > 2σ(*I*)
graphite	*R*_int_ = 0.040
φ and ω scans	θ_max_ = 25.0°, θ_min_ = 2.0°
Absorption correction: multi-scan (*SADABS*; Sheldrick, 1996)	*h* = −13→13
*T*_min_ = 0.720, *T*_max_ = 0.769	*k* = −10→24
8117 measured reflections	*l* = −15→15

### Refinement

**Table:**

Refinement on *F*^2^	Secondary atom site location: difference Fourier map
Least-squares matrix: full	Hydrogen site location: inferred from neighbouring sites
*R*[*F*^2^ > 2σ(*F*^2^)] = 0.071	H-atom parameters constrained
*wR*(*F*^2^) = 0.202	*w* = 1/[σ^2^(*F*_o_^2^) + (0.1348*P*)^2^] where *P* = (*F*_o_^2^ + 2*F*_c_^2^)/3
*S* = 1.04	(Δ/σ)_max_ < 0.001
3778 reflections	Δρ_max_ = 1.19 e Å^−^^3^
363 parameters	Δρ_min_ = −0.32 e Å^−^^3^
15 restraints	Absolute structure: Flack (1983), 957 Friedel pairs
Primary atom site location: structure-invariant direct methods	Flack parameter: −0.02 (3)

### Special details

**Table:**

Geometry. All e.s.d.'s (except the e.s.d. in the dihedral angle between two l.s. planes) are estimated using the full covariance matrix. The cell e.s.d.'s are taken into account individually in the estimation of e.s.d.'s in distances, angles and torsion angles; correlations between e.s.d.'s in cell parameters are only used when they are defined by crystal symmetry. An approximate (isotropic) treatment of cell e.s.d.'s is used for estimating e.s.d.'s involving l.s. planes.
Refinement. Refinement of *F*^2^ against ALL reflections. The weighted *R*-factor *wR* and goodness of fit *S* are based on *F*^2^, conventional *R*-factors *R* are based on *F*, with *F* set to zero for negative *F*^2^. The threshold expression of *F*^2^ > σ(*F*^2^) is used only for calculating *R*-factors(gt) *etc*. and is not relevant to the choice of reflections for refinement. *R*-factors based on *F*^2^ are statistically about twice as large as those based on *F*, and *R*- factors based on ALL data will be even larger.

### Fractional atomic coordinates and isotropic or equivalent isotropic displacement parameters (Å^2^)

**Table:**

	*x*	*y*	*z*	*U*_iso_*/*U*_eq_	
Cu1	0.47884 (7)	0.39140 (5)	0.30240 (6)	0.0528 (3)	
O1	0.3247 (4)	0.3954 (4)	0.2218 (4)	0.0629 (13)	
O2	0.1982 (6)	0.4622 (4)	0.1409 (6)	0.086 (2)	
O3	0.6863 (7)	0.7563 (4)	0.1644 (6)	0.093 (2)	
H3	0.6884	0.7738	0.1089	0.139*	
O4	0.6105 (4)	0.3954 (4)	0.4054 (4)	0.0615 (13)	
O5	0.7833 (6)	0.3774 (4)	0.5400 (5)	0.085 (2)	
O6	0.0460 (5)	0.3655 (3)	0.1065 (5)	0.091 (2)	
H30	0.0785	0.3555	0.0545	0.110*	
H31	0.0698	0.4039	0.1183	0.110*	
O7	0.1122 (9)	0.5336 (6)	0.9760 (9)	0.140 (4)	
H33	0.1504	0.5389	1.0343	0.169*	
H32	0.1276	0.4940	0.9671	0.169*	
N1	0.4673 (6)	0.4843 (5)	0.2928 (5)	0.048 (2)	
N3	0.4572 (7)	0.2951 (4)	0.3234 (6)	0.057 (2)	
N2	0.5696 (6)	0.3454 (4)	0.1729 (5)	0.064 (2)	
C1	0.2914 (8)	0.4502 (5)	0.1928 (7)	0.064 (2)	
C2	0.3727 (7)	0.5070 (4)	0.2167 (6)	0.0523 (19)	
H2	0.3323	0.5430	0.2447	0.063*	
C3	0.4188 (7)	0.5280 (5)	0.1168 (6)	0.058 (2)	
H3A	0.4647	0.4932	0.0938	0.070*	
H3B	0.3544	0.5350	0.0647	0.070*	
C4	0.4896 (7)	0.5882 (4)	0.1279 (5)	0.0502 (18)	
C5	0.4378 (8)	0.6477 (5)	0.1252 (8)	0.072 (3)	
H5	0.3583	0.6500	0.1152	0.086*	
C6	0.5015 (8)	0.7054 (5)	0.1372 (7)	0.070 (2)	
H6	0.4648	0.7453	0.1353	0.084*	
C7	0.6210 (9)	0.7016 (5)	0.1519 (6)	0.067 (2)	
C8	0.6746 (8)	0.6425 (6)	0.1572 (7)	0.070 (3)	
H8	0.7542	0.6405	0.1691	0.084*	
C9	0.6109 (8)	0.5849 (5)	0.1449 (6)	0.059 (2)	
H9	0.6479	0.5451	0.1479	0.071*	
C10	0.5227 (7)	0.5261 (5)	0.3509 (6)	0.055 (2)	
H10	0.5003	0.5692	0.3433	0.065*	
C11	0.6173 (7)	0.5113 (5)	0.4271 (6)	0.055 (2)	
C12	0.6726 (9)	0.5654 (5)	0.4794 (7)	0.075 (3)	
H12	0.6470	0.6075	0.4654	0.090*	
C13	0.7641 (9)	0.5534 (7)	0.5505 (8)	0.089 (3)	
H13	0.8008	0.5880	0.5857	0.107*	
C14	0.8038 (10)	0.4923 (6)	0.5719 (7)	0.079 (3)	
H14	0.8680	0.4864	0.6194	0.095*	
C15	0.7517 (8)	0.4403 (6)	0.5254 (6)	0.061 (2)	
C16	0.6550 (7)	0.4463 (5)	0.4466 (6)	0.056 (2)	
C17	0.8766 (10)	0.3668 (7)	0.6178 (10)	0.106 (4)	
H17A	0.9476	0.3727	0.5899	0.158*	
H17B	0.8728	0.3235	0.6435	0.158*	
H17C	0.8722	0.3971	0.6723	0.158*	
C18	0.4013 (8)	0.2711 (5)	0.3963 (7)	0.068 (2)	
H18	0.3718	0.2998	0.4408	0.081*	
C19	0.3855 (10)	0.2077 (6)	0.4090 (9)	0.084 (3)	
H19	0.3461	0.1933	0.4617	0.101*	
C20	0.4272 (10)	0.1631 (6)	0.3443 (10)	0.085 (3)	
H20	0.4154	0.1189	0.3515	0.101*	
C21	0.4880 (8)	0.1874 (5)	0.2676 (8)	0.067 (2)	
C22	0.5018 (7)	0.2544 (5)	0.2576 (7)	0.059 (2)	
C23	0.5601 (7)	0.2798 (5)	0.1781 (6)	0.054 (2)	
C24	0.6054 (9)	0.2391 (6)	0.1102 (8)	0.073 (3)	
C25	0.6605 (8)	0.2653 (7)	0.0366 (8)	0.088 (3)	
H25	0.6918	0.2384	−0.0093	0.106*	
C26	0.6710 (10)	0.3290 (8)	0.0287 (9)	0.092 (3)	
H26	0.7081	0.3465	−0.0233	0.110*	
C27	0.6258 (8)	0.3701 (6)	0.0993 (7)	0.080 (3)	
H27	0.6350	0.4147	0.0948	0.096*	
C28	0.5357 (9)	0.1457 (6)	0.1986 (10)	0.085 (3)	
H28	0.5291	0.1010	0.2037	0.103*	
C29	0.5923 (12)	0.1733 (7)	0.1232 (10)	0.092 (4)	
H29	0.6236	0.1457	0.0783	0.110*	

### Atomic displacement parameters (Å^2^)

**Table:**

	*U*^11^	*U*^22^	*U*^33^	*U*^12^	*U*^13^	*U*^23^
Cu1	0.0548 (5)	0.0557 (6)	0.0464 (5)	−0.0062 (6)	−0.0006 (3)	0.0017 (5)
O1	0.062 (3)	0.053 (3)	0.070 (3)	−0.016 (4)	−0.005 (2)	0.000 (4)
O2	0.070 (4)	0.092 (5)	0.089 (5)	−0.018 (4)	−0.025 (4)	0.032 (4)
O3	0.096 (5)	0.082 (5)	0.102 (5)	−0.038 (4)	0.023 (4)	−0.002 (4)
O4	0.066 (3)	0.059 (3)	0.055 (3)	−0.004 (4)	−0.009 (2)	−0.001 (4)
O5	0.086 (4)	0.090 (7)	0.072 (4)	0.003 (4)	−0.021 (3)	0.009 (4)
O6	0.108 (5)	0.077 (5)	0.084 (5)	−0.032 (4)	−0.008 (4)	0.007 (4)
O7	0.127 (7)	0.129 (9)	0.162 (9)	0.001 (7)	0.002 (6)	0.026 (8)
N1	0.052 (4)	0.054 (5)	0.037 (3)	−0.001 (3)	0.003 (3)	0.003 (3)
N3	0.057 (4)	0.056 (5)	0.057 (4)	−0.004 (4)	0.001 (3)	−0.005 (4)
N2	0.053 (4)	0.084 (6)	0.052 (4)	−0.003 (4)	−0.006 (3)	−0.002 (4)
C1	0.055 (5)	0.063 (6)	0.071 (5)	−0.014 (4)	−0.004 (4)	0.009 (5)
C2	0.056 (4)	0.056 (5)	0.044 (4)	0.000 (4)	0.001 (3)	0.007 (4)
C3	0.055 (4)	0.073 (6)	0.046 (4)	−0.013 (4)	0.002 (3)	0.005 (4)
C4	0.060 (5)	0.051 (5)	0.039 (4)	−0.008 (4)	0.004 (3)	0.006 (3)
C5	0.053 (5)	0.073 (7)	0.092 (7)	0.009 (5)	0.019 (4)	0.020 (6)
C6	0.074 (6)	0.057 (6)	0.082 (6)	−0.003 (5)	0.020 (5)	0.006 (5)
C7	0.089 (6)	0.062 (6)	0.054 (5)	−0.014 (5)	0.021 (4)	0.000 (4)
C8	0.051 (5)	0.083 (7)	0.071 (6)	−0.021 (5)	−0.012 (4)	0.007 (5)
C9	0.067 (5)	0.064 (6)	0.047 (5)	−0.004 (5)	0.005 (4)	0.001 (4)
C10	0.062 (5)	0.054 (5)	0.044 (4)	−0.014 (4)	−0.005 (4)	0.001 (4)
C11	0.056 (4)	0.062 (6)	0.047 (4)	−0.009 (4)	0.008 (3)	−0.002 (4)
C12	0.097 (7)	0.066 (6)	0.058 (5)	−0.014 (6)	−0.005 (5)	−0.010 (5)
C13	0.086 (7)	0.099 (9)	0.076 (7)	−0.021 (6)	−0.016 (5)	−0.018 (6)
C14	0.088 (7)	0.094 (8)	0.051 (5)	−0.015 (6)	−0.006 (5)	0.000 (5)
C15	0.057 (5)	0.081 (7)	0.043 (4)	−0.005 (5)	0.000 (3)	0.011 (5)
C16	0.053 (4)	0.075 (6)	0.039 (4)	−0.006 (4)	0.001 (3)	−0.002 (4)
C17	0.088 (7)	0.126 (12)	0.090 (7)	0.011 (7)	−0.037 (6)	−0.001 (7)
C18	0.069 (5)	0.076 (7)	0.059 (5)	−0.006 (5)	0.014 (4)	0.005 (5)
C19	0.088 (7)	0.083 (8)	0.083 (7)	−0.026 (6)	0.018 (5)	0.003 (6)
C20	0.088 (7)	0.061 (6)	0.101 (8)	−0.016 (6)	−0.004 (6)	−0.007 (6)
C21	0.067 (5)	0.050 (5)	0.077 (6)	−0.006 (5)	−0.016 (4)	−0.005 (5)
C22	0.053 (4)	0.063 (6)	0.054 (5)	−0.008 (4)	−0.017 (4)	−0.003 (4)
C23	0.049 (4)	0.062 (6)	0.047 (4)	0.001 (4)	−0.003 (3)	−0.008 (4)
C24	0.067 (6)	0.085 (7)	0.063 (5)	0.005 (5)	−0.007 (4)	−0.016 (5)
C25	0.066 (6)	0.121 (8)	0.075 (6)	0.022 (7)	0.001 (5)	−0.012 (7)
C26	0.081 (7)	0.132 (9)	0.066 (6)	0.025 (7)	0.022 (5)	0.015 (7)
C27	0.076 (5)	0.105 (10)	0.060 (5)	0.001 (6)	0.013 (4)	0.015 (6)
C28	0.083 (7)	0.061 (7)	0.108 (9)	−0.005 (5)	−0.006 (6)	−0.005 (6)
C29	0.092 (8)	0.092 (8)	0.088 (8)	0.012 (7)	−0.001 (6)	−0.041 (7)

### Geometric parameters (Å, °)

**Table:**

Cu1—N1	1.926 (10)	C8—H8	0.9300
Cu1—O4	1.935 (5)	C9—H9	0.9300
Cu1—O1	1.989 (5)	C10—C11	1.439 (11)
Cu1—N3	2.029 (9)	C10—H10	0.9300
Cu1—N2	2.325 (8)	C11—C12	1.429 (13)
O1—C1	1.243 (12)	C11—C16	1.428 (13)
O2—C1	1.245 (11)	C12—C13	1.363 (15)
O3—C7	1.365 (12)	C12—H12	0.9300
O3—H3	0.8200	C13—C14	1.363 (17)
O4—C16	1.267 (12)	C13—H13	0.9300
O5—C15	1.357 (14)	C14—C15	1.347 (15)
O5—C17	1.425 (12)	C14—H14	0.9300
O6—H30	0.8500	C15—C16	1.450 (12)
O6—H31	0.8500	C17—H17A	0.9600
O7—H33	0.8500	C17—H17B	0.9600
O7—H32	0.8501	C17—H17C	0.9600
N1—C10	1.280 (11)	C18—C19	1.337 (16)
N1—C2	1.482 (10)	C18—H18	0.9300
N3—C18	1.325 (13)	C19—C20	1.384 (17)
N3—C22	1.358 (13)	C19—H19	0.9300
N2—C27	1.339 (12)	C20—C21	1.400 (17)
N2—C23	1.361 (12)	C20—H20	0.9300
C1—C2	1.521 (12)	C21—C22	1.402 (14)
C2—C3	1.547 (11)	C21—C28	1.418 (16)
C2—H2	0.9800	C22—C23	1.422 (13)
C3—C4	1.493 (12)	C23—C24	1.382 (14)
C3—H3A	0.9700	C24—C25	1.346 (16)
C3—H3B	0.9700	C24—C29	1.379 (18)
C4—C5	1.370 (13)	C25—C26	1.327 (19)
C4—C9	1.418 (13)	C25—H25	0.9300
C5—C6	1.405 (14)	C26—C27	1.412 (17)
C5—H5	0.9300	C26—H26	0.9300
C6—C7	1.396 (14)	C27—H27	0.9300
C6—H6	0.9300	C28—C29	1.39 (2)
C7—C8	1.373 (15)	C28—H28	0.9300
C8—C9	1.404 (14)	C29—H29	0.9300
			
N1—Cu1—O4	92.8 (3)	C12—C11—C16	122.4 (8)
N1—Cu1—O1	82.6 (3)	C12—C11—C10	116.1 (9)
O4—Cu1—O1	166.9 (3)	C16—C11—C10	121.5 (8)
N1—Cu1—N3	167.6 (3)	C13—C12—C11	117.7 (10)
O4—Cu1—N3	92.8 (3)	C13—C12—H12	121.1
O1—Cu1—N3	89.6 (3)	C11—C12—H12	121.1
N1—Cu1—N2	113.2 (3)	C14—C13—C12	122.3 (11)
O4—Cu1—N2	97.8 (2)	C14—C13—H13	118.9
O1—Cu1—N2	95.3 (2)	C12—C13—H13	118.9
N3—Cu1—N2	77.1 (3)	C15—C14—C13	121.2 (10)
C1—O1—Cu1	115.8 (6)	C15—C14—H14	119.4
C7—O3—H3	109.5	C13—C14—H14	119.4
C16—O4—Cu1	126.2 (7)	C14—C15—O5	126.5 (8)
C15—O5—C17	115.1 (9)	C14—C15—C16	122.2 (10)
H30—O6—H31	101.8	O5—C15—C16	111.2 (9)
H33—O7—H32	98.7	O4—C16—C11	126.9 (7)
C10—N1—C2	118.6 (9)	O4—C16—C15	118.9 (9)
C10—N1—Cu1	127.3 (6)	C11—C16—C15	114.2 (8)
C2—N1—Cu1	113.6 (6)	O5—C17—H17A	109.5
C18—N3—C22	119.8 (9)	O5—C17—H17B	109.5
C18—N3—Cu1	123.1 (8)	H17A—C17—H17B	109.5
C22—N3—Cu1	117.1 (7)	O5—C17—H17C	109.5
C27—N2—C23	117.8 (9)	H17A—C17—H17C	109.5
C27—N2—Cu1	133.5 (7)	H17B—C17—H17C	109.5
C23—N2—Cu1	108.7 (6)	N3—C18—C19	123.1 (10)
O1—C1—O2	125.1 (9)	N3—C18—H18	118.5
O1—C1—C2	118.1 (7)	C19—C18—H18	118.5
O2—C1—C2	116.7 (9)	C18—C19—C20	120.6 (10)
N1—C2—C1	107.4 (7)	C18—C19—H19	119.7
N1—C2—C3	110.8 (6)	C20—C19—H19	119.7
C1—C2—C3	108.4 (7)	C19—C20—C21	117.3 (10)
N1—C2—H2	110.1	C19—C20—H20	121.4
C1—C2—H2	110.1	C21—C20—H20	121.4
C3—C2—H2	110.1	C22—C21—C20	119.8 (10)
C4—C3—C2	113.5 (7)	C22—C21—C28	118.6 (10)
C4—C3—H3A	108.9	C20—C21—C28	121.6 (10)
C2—C3—H3A	108.9	N3—C22—C21	119.5 (9)
C4—C3—H3B	108.9	N3—C22—C23	120.1 (9)
C2—C3—H3B	108.9	C21—C22—C23	120.4 (9)
H3A—C3—H3B	107.7	N2—C23—C24	122.2 (9)
C5—C4—C9	118.8 (8)	N2—C23—C22	117.1 (8)
C5—C4—C3	120.2 (8)	C24—C23—C22	120.7 (10)
C9—C4—C3	121.0 (8)	C25—C24—C23	118.7 (12)
C4—C5—C6	121.9 (8)	C25—C24—C29	123.8 (12)
C4—C5—H5	119.1	C23—C24—C29	117.5 (11)
C6—C5—H5	119.1	C26—C25—C24	120.9 (12)
C7—C6—C5	118.8 (9)	C26—C25—H25	119.5
C7—C6—H6	120.6	C24—C25—H25	119.5
C5—C6—H6	120.6	C25—C26—C27	119.9 (11)
O3—C7—C8	118.8 (9)	C25—C26—H26	120.1
O3—C7—C6	120.9 (10)	C27—C26—H26	120.1
C8—C7—C6	120.3 (9)	N2—C27—C26	120.6 (12)
C7—C8—C9	120.8 (9)	N2—C27—H27	119.7
C7—C8—H8	119.6	C26—C27—H27	119.7
C9—C8—H8	119.6	C29—C28—C21	118.2 (11)
C8—C9—C4	119.4 (10)	C29—C28—H28	120.9
C8—C9—H9	120.3	C21—C28—H28	120.9
C4—C9—H9	120.3	C24—C29—C28	124.4 (12)
N1—C10—C11	124.7 (9)	C24—C29—H29	117.8
N1—C10—H10	117.6	C28—C29—H29	117.8
C11—C10—H10	117.6		
			
C1—C2—C3—C4	173.6 (7)		

### Hydrogen-bond geometry (Å, °)

**Table:**

*D*—H···*A*	*D*—H	H···*A*	*D*···*A*	*D*—H···*A*
O6—H31···O2	0.85	1.93	2.684 (9)	148
O3—H3···O6^i^	0.82	2.53	2.843 (11)	104
O6—H30···O6^ii^	0.85	2.44	2.888 (11)	114
O7—H33···O2^iii^	0.85	2.15	2.721 (13)	125
C18—H18···O5^iv^	0.93	2.46	3.268 (13)	145

Symmetry codes: (i) *x*+1/2, *y*+1/2, *z*; (ii) −*x*, *y*, −*z*; (iii) *x*, *y*, *z*+1; (iv) −*x*+1, *y*, −*z*+1.
